# Molecular Characterization and Expression Analysis of the C-Type Lectin Domain Family 4 Member F in *Litopenaeus vannamei* against White Spot Syndrome Virus

**DOI:** 10.3390/ani14081137

**Published:** 2024-04-09

**Authors:** Qian Xue, Bingbing Yang, Kun Luo, Sheng Luan, Jie Kong, Xupeng Li, Xianhong Meng

**Affiliations:** 1 State Key Laboratory of Mariculture Biobreeding and Sustainable Goods, Yellow Sea Fisheries Research Institute, Chinese Academy of Fishery Sciences, Qingdao 266071, China; 13011754639@163.com (Q.X.); nmzl19951209@163.com (B.Y.); luokun@ysfri.ac.cn (K.L.); luansheng@ysfri.ac.cn (S.L.); kongjie@ysfri.ac.cn (J.K.); 2 School of Fishery, Zhejiang Ocean University, Zhoushan 316021, China; 3 Laboratory for Marine Fisheries Science and Food Production Processes, Qingdao Marine Science and Technology Center, Qingdao 266237, China

**Keywords:** viral disease control, innate immunity, *LvCLEC4F*, WSSV, gene expression, RNAi

## Abstract

**Simple Summary:**

Outbreaks of white spot disease in Pacific white shrimp pose a significant threat to the major shrimp farming industry. Understanding the mechanisms of the shrimp against the causative virus is crucial. C-type lectins are important pattern recognition receptors that can be involved in the response against viral infections. This study identified the C-type lectin domain family 4 member F in shrimp as an important receptor gene that could promote replication of the causative virus and affect the survival rate of shrimp. This study will provide a theoretical basis for understanding the resistance mechanisms of shrimp against the virus.

**Abstract:**

White spot disease (WSD) outbreaks pose a significant threat to the Pacific white shrimp (*Litopenaeus vannamei*) farming industry. The causative agent is the white spot syndrome virus (WSSV). There are no effective treatments for WSD so far. Therefore, understanding the resistance mechanisms of *L*. *vannamei* against the WSSV is crucial. C-type lectins (CTLs) are important pattern recognition receptors (PRRs) that promote agglutination, phagocytosis, encapsulation, bacteriostasis, and antiviral infections. This study cloned the C-type lectin domain family 4 member F (*LvCLEC4F*) from *L*. *vannamei*. *LvCLEC4F* contains a 492 bp open reading frame (ORF) encoding a protein of 163 amino acids, including a carbohydrate recognition domain (CRD). Following a challenge with the WSSV, the expression profile of *LvCLEC4F* was significantly altered. Using RNA interference (RNAi) technology, it was found that *LvCLEC4F* promotes WSSV replication and affects the expression levels of genes related to the regulation of apoptosis, signaling and cellular stress response, and immune defense. Meanwhile, the hemolymph agglutination phenomenon in vivo was weakened when *LvCLEC4F* was knocked down. These results indicated that *LvCLEC4F* may play an important role in the interaction between *L*. *vannamei* and WSSV.

## 1. Introduction

The Pacific white shrimp *Litopenaeus vannamei* has become a major shrimp farming species worldwide because of its rapid growth, strong disease resistance, and economic benefits [1]. With the expansion of aquaculture, sudden outbreaks of bacterial, fungal, and viral diseases pose a significant threat to the global shrimp farming industry. White spot disease (WSD) is one of the most serious diseases in shrimp, and the causative agent is the white spot syndrome virus (WSSV). WSSV, an enveloped double-stranded DNA virus, ranges from 250 to 380 nm in length and 80 to 120 nm in diameter and is classified in the genus *Whispovirus* of the family *Nimaviridae* [2]. Within 7–10 days, the mortality rate of shrimp can reach 100% following WSSV infection [3,4]. Despite considerable research into treatment approaches and various methods to prevent WSD in recent years, the development of effective control strategies and treatments remains an ongoing challenge [5]. Therefore, understanding the resistance mechanisms of *L*. *vannamei* against the WSSV is crucial.

Invertebrates lack an acquired immune system and rely mainly on the innate immune system to resist foreign bodies and pathogens [6]. Following bacterial or viral infection, host pattern recognition receptors (PRRs) recognize pathogen-associated molecular patterns (PAMPs), which trigger the initiation of innate immune defense [7,8]. Various PRRs have been identified in invertebrates, including toll-like receptors (TLRs), scavenger receptors (SRs), gram-negative binding proteins (GNBPs), and C-type lectins (CTLs). PRRs elicit various immune responses. Therefore, functional research on PRRs has attracted widespread attention [9].

CTLs are important PRRs and are widespread in both vertebrates and invertebrates. CTLs typically contain one or more carbohydrate recognition domains (CRDs). The CRD includes 110–130 amino acids and has a conserved structural motif arranged in two protein loops stabilized by two disulfide bridges [10]. CTLs specifically bind to glycoproteins on the surface of pathogens via CRDs, which triggers a series of immune responses [11,12]. CTLs mediate intercellular interactions, regulate cytokine expression, promote phagocytosis and encapsulation, activate inflammatory responses, and play important roles in cell apoptosis [13,14,15].

CTLs play important roles in the invertebrate immune processes. Stomach virus-associated CTLs capture viral protein post-WSSV infection and present it to receptors on the surfaces of related shrimp host cells, facilitating WSSV infection in shrimp [16]. When recombinant CTL (*rLv*Lec) was injected into *L*. *vannamei* infected with *Vibrio*, *Lv*Lec regulated the immune response of hemocytes via the cGMP-PKA signaling pathway [17]. LvCTL5 from *L*. *vannamei* was found to have a direct bacteriostatic and immunoregulatory function [18]. A shrimp CTL, as the PRR of bacteria and the ligand of the dome, mediates the activation of the Jak/STAT pathway [19]. LvCTL 4.2 in *L*. *vannamei* inhibited *Vibrio* but facilitated WSSV infection [20].

This study identified the C-type lectin domain family 4 member F (*LvCLEC4F*) in *L*. *vannamei*. To determine whether *LvCLEC4F* is involved in the response of *L*. *vannamei* to WSSV infection, the expression profile of *LvCLEC4F* was determined in the hepatopancreas, gill, muscle, and eyestalk of healthy and WSSV-infected *L*. *vannamei*. Using RNA interference (RNAi) technology to knockdown the expression of *LvCLEC4F*, the specific function of *LvCLEC4F* in the anti-WSSV response was analyzed. Hemolymph agglutination in vivo of *L*. *vannamei*, WSSV viral load, *L*. *vannamei* survival rate, and the expression levels of genes related to the regulation of apoptosis, signaling and cellular stress response, and immune defense were detected.

## 2. Materials and Methods

### 2.1. Experimental Animals

All pathogenic-free *L*. *vannamei* were obtained from BLUP Aquabreed Co., Ltd. (Weifang, China). Samples of *L*. *vannamei* were randomly selected and subjected to diagnostic screening for seven pathogens, including WSSV, acute hepatopancreatic necrosis disease-causing *Vibrio parahaemolyticus* (*Vp*_AHPND_), covert mortality nodavirus (CMNV), infectious hypodermal and hematopoietic necrosis virus (IHHNV), infectious myonecrosis virus (IMNV), Decapod iridescent virus 1 (DIV1), and Enterocytozoon hepatopenaei (EHP). No pathogens were identified. Experiments were conducted at the Aquatic Genetic Breeding Center, Yellow Sea Fisheries Research Institute, Chinese Academy of Fishery Sciences (Qingdao, China). Before beginning animal experiments, *L*. *vannamei* were acclimatized in sterile seawater (salinity 29‰, temperature 26 ± 1°C) for three days. *L*. *vannamei* were subjected to the same daily management, including changing water once a day (each time 1/2 vol), supplying sufficient dissolved oxygen, and providing a consistent diet of commercial feed. *L*. *vannamei* with 2.5–3.5 g body weight and 6.0–6.6 cm body length were used to clone the *LvCLEC4F* sequence and analyze the *LvCLEC4F* expression profiles post-WSSV infection. *L*. *vannamei* with 2.7–3.3 g body weight and 5.5–6.3 cm body length were used for the RNAi experiment.

### 2.2. RNA Extraction and cDNA Synthesis

Total RNA was extracted using the RNA-Easy isolation reagent (Vazyme Biotech Co., Ltd., Nanjing, China) following the manufacturer’s instructions. The quality and concentration of RNA were assessed by 1% agarose gel electrophoresis using a NanoPhotometer^®^ spectrophotometer (IMPLEN, Munchen, Germany). For the real-time PCR (RT-PCR) assay, cDNA was synthesized from total RNA using a HiScript^®^ III RT SuperMix for qPCR (+gDNA wiper) kit (Vazyme Biotech Co., Ltd., Nanjing, China) following the manufacturer’s instructions.

### 2.3. Molecular Cloning and Sequencing of LvCLEC4F

Based on the reference sequence of *LvCLEC4F* in the NCBI database (XM_027356524.1), primer3 (https://primer3.ut.ee/, accessed on 26 March 2023) was used to design the primers (LvCLEC4F-F and LvCLEC4F-R) (Table 1) for cDNA sequence amplification of *LvCLEC4F*. The cDNA solution was acquired from the hepatopancreas in *L*. *vannamei*. The ORF sequence of *LvCLEC4F* was amplified using Quick Taq HS DyeMix (TOYOBO, Shanghai, China). PCR was performed in a 50 μL volume containing 4 μL cDNA solution (100 ng/μL), 25 μL 2x Quick Taq HS DyeMix, and 1 μL of each primer (10 μM). The PCR reaction was performed as follows: 94 °C for 2 min; 94 °C for 30 s, 55 °C for 30 s, and 68 °C for 1 min for 35 cycles; and it was extended at 68 °C for 5 min. PCR products were sequenced by Sangon Biotech Co., Ltd. (Shanghai, China).

### 2.4. Bioinformatics Analysis

The ORF sequence of *LvCLEC4F* was translated into an amino acid sequence using EditSeq 7.1 software (https://www.dnastar.com/, accessed on 18 April 2023). Protein molecular weight (MW) and the theoretical isoelectric point (PI) instability coefficient were predicted using the ExPASy-ProtParam tool (https://web.expasy.org/protparam/, accessed on 18 April 2023). The transmembrane region of the protein was analyzed using TMHMM 2.0 software (http://www.cbs.dtu.dk/services/TMHMM/, accessed on 18 April 2023). SignalP 4.1 software (https://services.healthtech.dtu.dk/services/SignalP-4.1/, accessed on 20 April 2023) was used to predict the signal peptide. Glycosylation sites were analyzed using NetNGlyc 1.0 online software (https://services.healthtech.dtu.dk/services/NetNGlyc-1.0/, accessed on 21 April 2023). The phosphorylation site analysis used NetPhos 3.1 software (https://services.healthtech.dtu.dk/services/NetPhos-3.1/, accessed on 21 April 2023). The SMART online tool (https://smart.embl.de/, accessed on 21 April 2023) was used for protein functional domain prediction analysis. The secondary structure of the protein was predicted using SOPMA software (https://npsa-prabi.ibcp.fr/, accessed on 21 April 2023). Alignment with homologous sequences from other species was performed using DNAMAN 6.0 (https://www.lynnon.com/, accessed on 28 April 2023). The phylogenetic tree for LvCLEC4F was constructed using the neighbor-joining (NJ) method of MEGA 7.0 (https://www.megasoftware.net/, accessed on 28 April 2023).

### 2.5. WSSV Challenge Experiment

The experiment was divided into three groups, with fifty shrimp per group. To detect the distribution of *LvCLEC4F*, the hepatopancreas, gill, muscle, and eyestalk of healthy *L*. *vannamei* were collected. Three shrimp from every group were taken. The remaining *L*. *vannamei* were individually orally infected with the WSSV bait. The viral load of the WSSV bait was 1 × 10^7^ copies. The hepatopancreas, gill, muscle, and eyestalk of *L*. *vannamei* at 24, 48, 72, 96, 144, 192, and 228 h post-WSSV infection were collected and stored in RNAstore reagent (Tiangen Biotech Co., Ltd., Beijing, China) at −80 °C. Three individual shrimp from each group were taken at every sampling time. The collected samples were used for total RNA extraction and cDNA synthesis to assess the expression level of *LvCLEC4F*.

### 2.6. RT-PCR

The expression level of *LvCLEC4F* was detected using an RT-PCR assay with SYBR Green Real-time PCR Master Mix (TOYOBO, Shanghai, China) and the Applied Biosystems^TM^ QuantStudio 1 Real-Time PCR quantifier (Applied Biosystems, Foster City, CA, USA) with primers (qLvCLEC4F-F, qLvCLEC4F-R, 18S-F, and 18S-R) (Table 1). 18S ribosomal RNA (18S rRNA) was used as an internal control. RT-PCR was performed in a 20 μL volume containing 10 μL SYBR Green Realtime PCR Master Mix, 0.8 μL of each primer (10 μM), and 2 μL cDNA solution (100 ng/μL). RT-PCR was performed as follows: 95 °C for 60 s; 95 °C for 15 s, 60 °C for 15 s, and 72 °C for 45 s for 40 cycles. All experiments were performed in triplicate, and the data were analyzed using the 2^–ΔΔCt^ method. An unpaired two-tailed t-test was used to compare the results.

### 2.7. Synthesis of Double-Stranded RNA

Double-stranded RNAs (dsRNAs) targeting *LvCLEC4F* (designated *dsLvCLEC4F*) were synthesized by in vitro transcription. dsRNAs targeting GFP (designated *dsGFP*) were also synthesized and used as negative controls. The DNA template for *dsLvCLEC4F* preparation was generated by PCR using the primers dsLvCLEC4F-Fi and dsLvCLEC4F-Ri (Table 1), with cDNA from the hepatopancreas of *L*. *vannamei* as a template. The DNA template for *dsGFP* preparation was generated by PCR using the primers dsGFP-Fi and dsGFP-Ri (Table 1), with the plasmid pET28a containing the GFP sequence as a template. The PCR products contained the T7 promoter sequence. According to the manufacturer’s instructions, dsRNAs were synthesized using a T7 in vitro transcription kit (Takara, Dalian, China). The in vitro transcription was performed in a 20 μL volume, which contained 2 μL 10 × transcription buffer, 2 μL ATP solution, 2 μL GTP solution, 2 μL CTP solution, 2 μL UTP solution, 0.5 μL Rnase inhibitor, 2 μL T7 RNA polymerase, and 3 μL DNA templates (1 μg). The reaction mixture was incubated at 42 °C for 2 h and then was incubated at 37 °C for 30 min after being added to 2 μL of Rnase-free Dnase I. The dsRNAs were stored at −80 °C.

### 2.8. Knockdown of LvCLEC4F by dsRNA

To assess the RNAi-mediated knockdown efficiency of the dsRNAs, 60 *L*. *vannamei* were randomly divided into two groups: *dsGFP*+WSSV and *dsLvCLEC4F*+WSSV. *L*. *vannamei* was injected at the third abdominal segment with *dsGFP* (3 μg/g shrimp) (*dsGFP*+WSSV group) and ds*LvCLEC4F* (3 μg/g shrimp) (*dsLvCLEC4F*+WSSV group), respectively. A total of 24 h after the first injection, the same dose of dsRNAs was injected again to enhance RNAi efficiency, followed by the injection of WSSV virus suspension (4.7 × 10^6^ copies) in a 20 μL volume for the *dsGFP*+WSSV and *dsLvCLEC4F*+WSSV groups. The hepatopancreas of *L*. *vannamei* in the two groups were collected at 36 and 48 h post-WSSV injection. The expression level of *LvCLEC4F* in the hepatopancreas was determined using RT-PCR with three replicates.

### 2.9. Survival Rate Analysis after LvCLEC4F Knockdown

The healthy *L*. *vannamei* were randomly divided into four groups (thirty individual shrimp per group), named the *dsLvCLEC4F*+WSSV group, *dsGFP*+WSSV group (as control), PBS group (as control), and WSSV group (as control). *L*. *vannamei* was injected at the third abdominal segment with ds*LvCLEC4F* (3 μg/g shrimp) (*dsLvCLEC4F*+WSSV group), *dsGFP* (3 μg/g shrimp) (*dsGFP*+WSSV group), or 1 × PBS (PBS group), respectively. A total of 24 h after the first injection, the same dose of dsRNAs was injected again, followed by the injection of WSSV virus suspension (4.7 × 10^6^ copies) in a 20 μL volume for the *dsLvCLEC4F*+WSSV, *dsGFP*+WSSV, and WSSV groups. The survival rates of *L*. *vannamei* in four groups were determined at different time points (0, 12, 24, 36, 48, 72, and 96 h) after WSSV injection. The survival rates between different groups of *L*. *vannamei* were tested for statistical significance using the log-rank test. Statistical analyses were performed using GraphPad Prism software (version 8.0; https://www.graphpad.com/, accessed on 12 September 2023).

### 2.10. Investigating Immune Response and Gene Expression after LvCLEC4F Knockdown

#### 2.10.1. Experimental Setting

Hemolymph agglutination in vivo, the viral load of WSSV, and the gene expression related to the innate immunity of *L*. *vannamei* were assessed after *LvCLEC4F* knockdown. *L*. *vannamei* were randomly divided into four groups (thirty individual shrimp per group), named the *dsLvCLEC4F*+WSSV group, *dsGFP*+WSSV group, PBS group, and WSSV group. *L*. *vannamei* was injected at the third abdominal segment with *dsLvCLEC4F* (3 μg/g shrimp) (*dsLvCLEC4F*+WSSV group), *dsGFP* (3 μg/g shrimp) (*dsGFP*+WSSV group), or 1 × PBS (PBS group), respectively. A total of 24 h after the first injection, the same dose of dsRNAs was injected again, followed by the injection of WSSV virus suspension (4.7 × 10^6^ copies) in a 20 μL volume for the *dsLvCLEC4F*+WSSV, *dsGFP*+WSSV, and WSSV group. The hepatopancreas and muscle of *L*. *vannamei* were collected at 0, 36, 48, and 72 h post-WSSV infection and stored at −80 °C. The hemolymph of *L*. *vannamei* was collected at 48 h post-WSSV infection. All experiments were performed in triplicate.

#### 2.10.2. Detection of Hemolymph Agglutination

The hemolymph agglutination phenomenon in vivo at 48 h post-WSSV infection was observed using an optical microscope. All experiments were performed in triplicate.

#### 2.10.3. The WSSV Viral Load Analysis with TaqMan RT-PCR

DNA was extracted from the muscle of *L*. *vannamei* using a TIANamp Marine Animals DNA Kit (Tiangen Biotech Co., Ltd., Beijing, China). The quality and concentration of DNA were assessed by 1% agarose gel electrophoresis and using a NanoPhotometer^®^ spectrophotometer (IMPLEN, Munchen, Germany). The copy number of WSSV in the muscle of *L*. *vannamei* was detected using TaqMan RT-PCR with an Applied Biosystems^TM^ QuantStudio 1 Real-Time PCR Quantifier (Applied Biosystems, Foster City, CA, USA) and the THUNDERBIRD^TM^ Probe qPCR Mix kit (TOYOBO, Shanghai, China) with primers WSSV-F and WSSV-R and the WSSV probe (Table 1) [21]. TaqMan RT-PCR was performed in a 20 μL volume containing 10 μL THUNDERBIRD Probe qPCR Mix, 0.6 μL of each primer (10 μM), 0.4 μL WSSV probe (10 μM), 0.1 μL ROX reference dye, and 2 μL DNA template. TaqMan RT-PCR was performed as follows: 95 °C for 30 s, 40 cycles of 95 °C for 5 s, and 60 °C for 34 s. All experiments were performed in triplicate. The WSSV viral load in *L*. *vannamei* was measured by quantifying the concentration of WSSV DNA.

#### 2.10.4. Gene Expression Analysis

To further confirm the role of the *LvCLEC4F* in anti-WSSV innate immunity, RT-PCR was used to examine the expression levels of genes related to innate immunity, including B cell leukemia (*Bcl-2*) (XM_027353493.1), caspase 3 (KC660103.1), caspase 8 (XM_027383230.1), mitogen-activated protein kinase p38b-like (*p38MAPK*) (XM_027367740.1), and lysozyme (*Lyz*) (AY170126) in the above-mentioned RNAi assay. Total RNA was extracted from the hepatopancreas of *L*. *vannamei* infected with WSSV at different time points (0, 36, 48, and 72 h) in the *dsGFP*+WSSV and *dsLvCLEC4F*+WSSV groups. The RNA extraction, cDNA synthesis, and RT-PCR methods are as described above. Gene expression was detected with primers (Bcl-2-F, Bcl-2-R, caspase 3-F, caspase 3-R, caspase 8-F, caspase 8-R, p38MAPK-F, p38MAPK-R, Lyz-F, Lyz-R, 18S-F, and 18S-R) (Table 1) with three replicates.

## 3. Results

### 3.1. Characterization of LvCLEC4F

**Figure 1:**
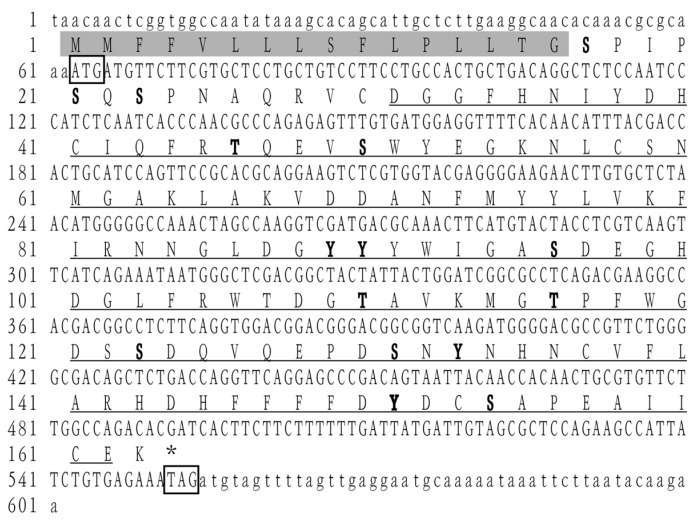
cDNA and amino acid sequence of *LvCLEC4F*. The start and stop codons are indicated by boxes. The predicted carbohydrate recognition domain (CRD) is indicated by an underline. The predicted phosphorylation sites are indicated in bold font. The predicted signal peptide structure is indicated by a gray shadow.

The complete ORF sequence of *LvCLEC4F* was 492 bp, encoding 163 amino acids (Figure 1). The MW was 18.70 kDa, the theoretical PI was 4.88, and the instability index was 40.53, which was classified as an unstable protein. There were 20 negatively and 11 positively charged amino acid residues. The aliphatic index was 65.21, and the average hydrophilicity was -0.273. This protein was predicted to contain eight serine (Ser), three threonine (Thr), and four tyrosine (Tyr) phosphorylation sites. A signal peptide consisting of 16 amino acids was present at the N-terminus. The CRD was located at positions 30–162 (Figure 1). Among the predicted secondary structures of LvCLEC4F after the removal of the signal peptide, the α-helix (26 amino acids) accounted for 17.69%, the β-strand (40 amino acids) accounted for 27.21%, the beta-turn (12 amino acids) accounted for 8.16%, and the random coil (69 amino acids) accounted for 46.94%.

### 3.2. Multiple Alignments and Phylogenetic Analysis

**Figure 2:**
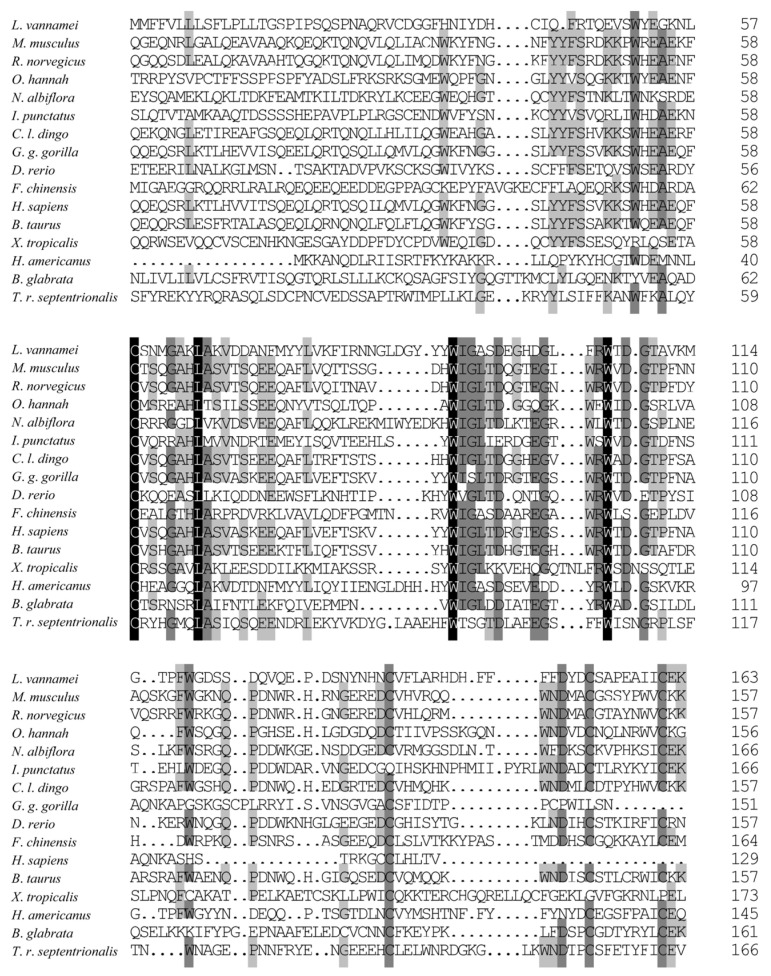
The amino acid sequence alignment of CLEC4F. One hundred percent identical residues are indicated by a black shadow. Seventy-five percent identical residues are indicated by a dark gray shadow. Fifty percent identical residues are indicated by a light gray shadow. The GenBank accession numbers of CLEC4F are as follows: *L*. *vannamei* (XP_027212325.1), *Rattus norvegicus* (NP_446205.1), *Canis lupus dingo* (XP_025326743.1), *Gorilla gorilla gorilla* (XP_055234888.1), *Danio rerio* (XP_009299422.1), *Fenneropenaeus chinensis* (XP_047498904.1), *Homo sapiens* (KAI4034935.1), *Bos taurus* (XP_027410747.1), *Xenopus tropicalis* (XP_031750858.1), *Homarus americanus* (XP_042233235.1), *Toxorhynchites rutilus septentrionalis* (XP_055620181.1), *Mus musculus* (NP_058031.2), *Ophiophagus hannah* (ETE57245.1), *Nibea albiflora* (KAG8004890.1), *Ictalurus punctatus* (NP_001187725.1), *Biomphalaria glabrata* (XP_055887399.1).

To further investigate the amino acid sequence similarity between LvCLEC4F and CLEC4F in other species, several vertebrates and invertebrates were selected for multiple comparisons of CLEC4F. Sequence conservation of the CLEC4F protein is relatively low, with significant differences in amino acid sequences among different species. The homology between LvCLEC4F and CLEC4F of the American lobster *Homarus americanus* was the highest, with a similarity of 49.12%. This was followed by some vertebrates (40.30–21.93%) and invertebrates (23.73–20.31%) (Figure 2).

**Figure 3:**
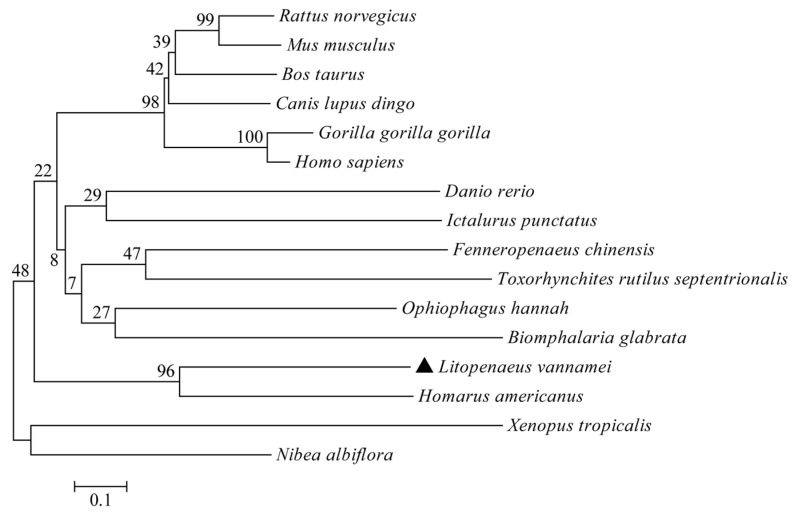
Phylogenetic tree analysis of CLEC4F. The GenBank accession numbers of CLEC4F sequences are identical to those listed in Figure 2. The LvCLEC4F marker of *L*. *vannamei* is ▲.

A phylogenetic evolutionary analysis was conducted to study the evolutionary relationship between LvCLEC4F and the selected vertebrates and invertebrates further. The phylogenetic tree was consistent with the taxonomic status of the species. *L*. *vannamei* clustered preferentially with *H*. *americanus* (Figure 3).

### 3.3. The Expression Profiles of LvCLEC4F Post-WSSV Infection

**Figure 4:**
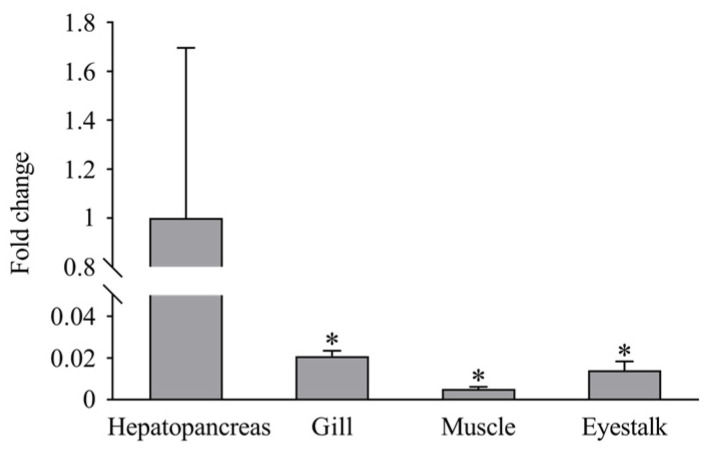
Expression profiles of *LvCLEC4F* in the hepatopancreas, gill, muscle, and eyestalk of healthy *L*. *vannamei*. *: *p* < 0.05.

RT-PCR demonstrated that *LvCLEC4F* was expressed in the hepatopancreas, gill, muscle, and eyestalk of healthy *L*. *vannamei*. The expression level of *LvCLEC4F* in the hepatopancreas was significantly higher than in the gill, muscle, and eyestalk. The expression level of *LvCLEC4F* in the gill was approximately 0.021-fold (*p* < 0.05) the level in the hepatopancreas. The expression level of *LvCLEC4F* in the muscle was approximately 0.005-fold (*p* < 0.05) the level in the hepatopancreas. The expression level of *LvCLEC4F* in the eyestalk was approximately 0.014-fold (*p* < 0.05) the level in the hepatopancreas (Figure 4).

**Figure 5:**
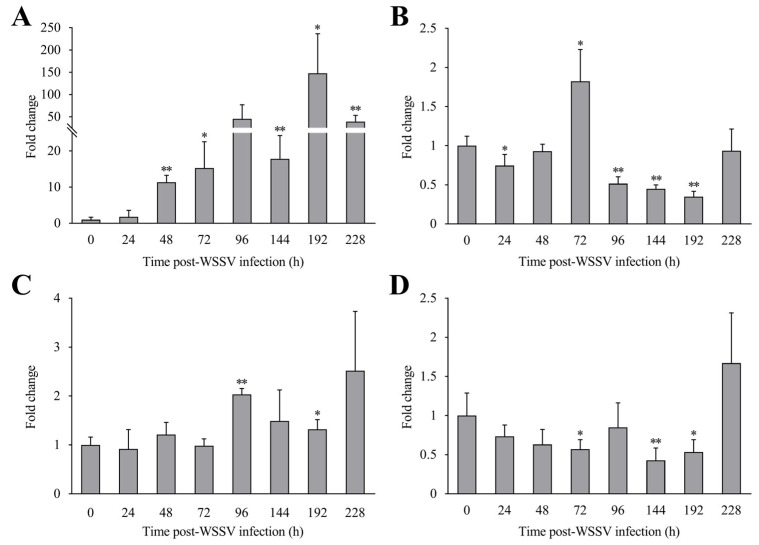
Expression profiles of *LvCLEC4F* in the hepatopancreas, gill, muscle, and eyestalk of *L*. *vannamei* post-WSSV infection. (**A**) The expression level of *LvCLEC4F* in the hepatopancreas at different time points post-WSSV infection. (**B**) The expression level of *LvCLEC4F* in the gill at different time points post-WSSV infection. (**C**) The expression level of *LvCLEC4F* in the muscle at different time points post-WSSV infection. (**D**) The expression level of *LvCLEC4F* in the eyestalk at different time points post-WSSV infection. *: *p* < 0.05, **: *p* < 0.01.

Post-WSSV infection, the expression levels of *LvCLEC4F* in the hepatopancreas of *L*. *vannamei* at 24, 48, 72, 96, 144, 192, and 228 h were approximately 1.78-fold, 11.32-fold (*p* < 0.01), 15.29-fold (*p* < 0.05), 45.19-fold, 17.82-fold (*p* < 0.01), 147.69-fold (*p* < 0.05), and 39.58-fold (*p* < 0.01) the level in the control, respectively (Figure 5A). The expression levels of *LvCLEC4F* in the gill at 24, 48, 72, 96, 144, 192, and 228 h were approximately 0.75-fold (*p* < 0.05), 0.93-fold, 1.82-fold (*p* < 0.05), 0.52-fold (*p* < 0.01), 0.45-fold (*p* < 0.01), 0.35-fold (*p* < 0.01), and 0.94-fold the level in the control, respectively (Figure 5B). The expression levels of *LvCLEC4F* in the muscle at 24, 48, 72, 96, 144, 192, and 228 h were approximately 0.92-fold, 1.21-fold, 0.99-fold, 2.04-fold (*p* < 0.01), 1.49-fold, 1.32-fold (*p* < 0.05), and 2.51-fold the level in the control, respectively (Figure 5C). The expression levels of *LvCLEC4F* in the eyestalk at 24, 48, 72, 96, 144, 192, and 228 h were approximately 0.74-fold, 0.63-fold, 0.57-fold (*p* < 0.05), 0.85-fold, 0.43-fold (*p* < 0.01), 0.53-fold (*p* < 0.05), and 1.67-fold the level in the control, respectively (Figure 5D).

### 3.4. WSSV Infection Was Suppressed after Knocking down LvCLEC4F

**Figure 6:**
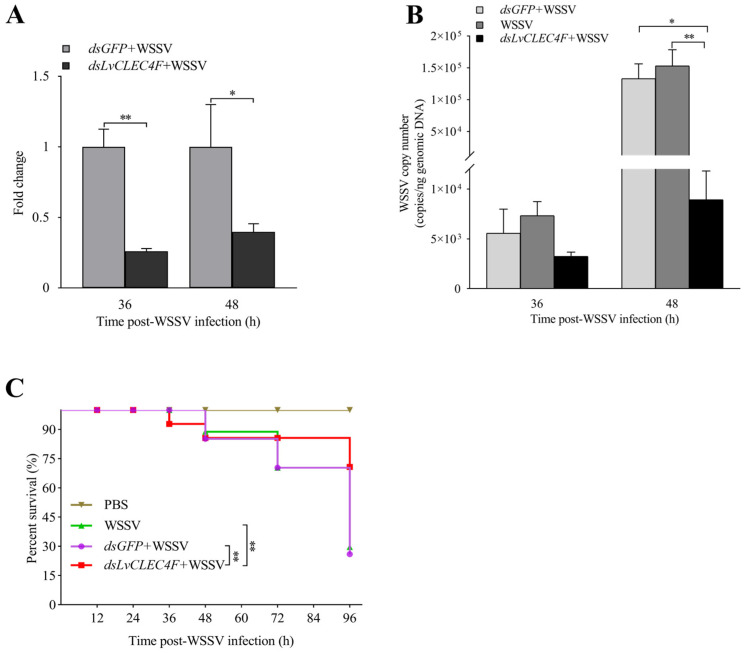
WSSV replication was suppressed after knocking down *LvCLEC4F*. (**A**) The knockdown efficiency of *LvCLEC4F* in the hepatopancreas at 36 and 48 h post-WSSV infection. (**B**) The WSSV viral load in the *dsGFP*+WSSV group (as control), the WSSV group (as control), and the *dsLvCLEC4F*+WSSV group after knocking down *LvCLEC4F*. (**C**) The survival curve of *L*. *vannamei* post-WSSV infection. *: *p* < 0.05, **: *p* < 0.01.

An RT-PCR assay was conducted to confirm RNAi efficiency. The results showed that the *LvCLEC4F* expression level in the *dsLvCLEC4F*+WSSV group was reduced by approximately 73.94% and 60.18% at 36 and 48 h post-WSSV infection, respectively, compared with that in the *dsGFP*+WSSV group (Figure 6A), indicating the successful in vivo knockdown of *LvCLEC4F*.

A TaqMan RT-PCR assay was performed to investigate the characteristics of WSSV replication in the muscle of *L*. *vannamei* after knocking down *LvCLEC4F*. The results showed that the WSSV copy number in *L*. *vannamei* in the *dsGFP*+WSSV group was 5.57 × 10^3^ copies/ng at 36 h post-WSSV infection. The WSSV copy number in *L*. *vannamei* in the WSSV group was 7.34 × 10^3^ copies/ng at 36 h post-WSSV infection. The WSSV copy number in *L*. *vannamei* in the *dsLvCLEC4F*+WSSV group was 3.25 × 10^3^ copies/ng at 36 h post-WSSV infection (Figure 6B). The WSSV copy number in *L*. *vannamei* in the *dsGFP*+WSSV group was 1.33 × 10^5^ copies/ng at 48 h post-WSSV infection. The WSSV copy number in *L*. *vannamei* in the WSSV group was 1.53 × 10^5^ copies/ng at 48 h post-WSSV infection. The WSSV copy number in *L*. *vannamei* in the *dsLvCLEC4F*+WSSV group was 8.93 × 10^3^ copies/ng at 48 h post-WSSV infection. The WSSV copy number in *L*. *vannamei* in the *dsLvCLEC4F*+WSSV group was significantly lower (0.067-fold) (*p* < 0.05) than that in the *dsGFP*+WSSV group and significantly lower (0.058-fold) (*p* < 0.01) than that in the WSSV group at 48 h post-WSSV infection (Figure 6B). Subsequently, the survival rates of *L*. *vannamei* at 0, 12, 24, 36, 48, 72, and 96 h post-WSSV infection were analyzed. The survival rate of *L*. *vannamei* in the PBS group was 100%. There was no significant difference in the survival rate of *L*. *vannamei* between the *dsGFP*+WSSV and WSSV groups. The survival rate of *L*. *vannamei* in the *dsLvCLEC4F*+WSSV group was significantly higher than that in the *dsGFP*+WSSV and WSSV groups (*p* < 0.01) (Figure 6C).

### 3.5. Hemolymph Agglutination In Vivo Assay

**Figure 7:**
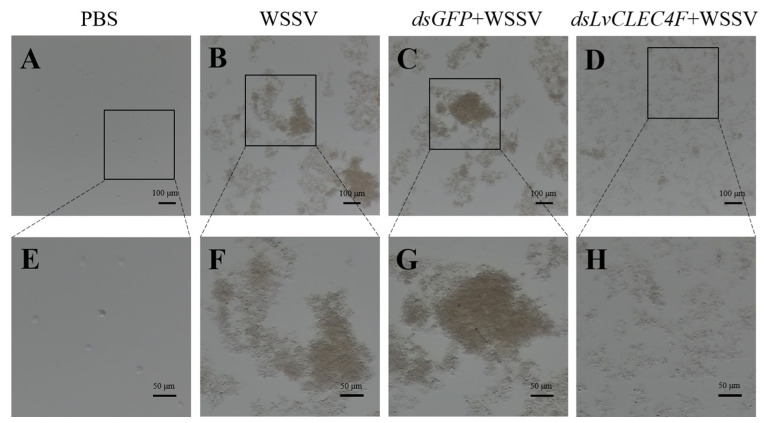
The observation of hemolymph agglutination in *L*. *vannamei* following WSSV infection after knocking down *LvCLEC4F*. (**A**) PBS group (scale bar = 100 μm). (**B**) WSSV group (scale bar = 100 μm). (**C**) *dsGFP*+WSSV group (scale bar = 100 μm). (**D**) *dsLvCLEC4F*+WSSV group (scale bar = 100 μm). (**E**) PBS group (scale bar = 50 μm). (**F**) WSSV group (scale bar = 50 μm). (**G**) *dsGFP*+WSSV group (scale bar = 50 μm). (**H**) *dsLvCLEC4F*+WSSV group (scale bar = 50 μm).

Optical microscopy was performed to investigate the effect of *LvCLEC4F* knockdown on hemolymph agglutination in *L*. *vannamei*. The results showed that there was no apparent agglutination in the PBS group (Figure 7A,E). The hemolymph agglutination phenomenon was weakened in the *dsLvCLEC4F*+WSSV group (Figure 7D,H) compared with the WSSV (Figure 7B,F) and *dsGFP*+WSSV (Figure 7C,G) groups after knocking down *LvCLEC4F* at 48 h post-WSSV infection.

### 3.6. The Expression Profiles of Genes Related to Innate Immunity after Knocking down LvCLEC4F

**Figure 8:**
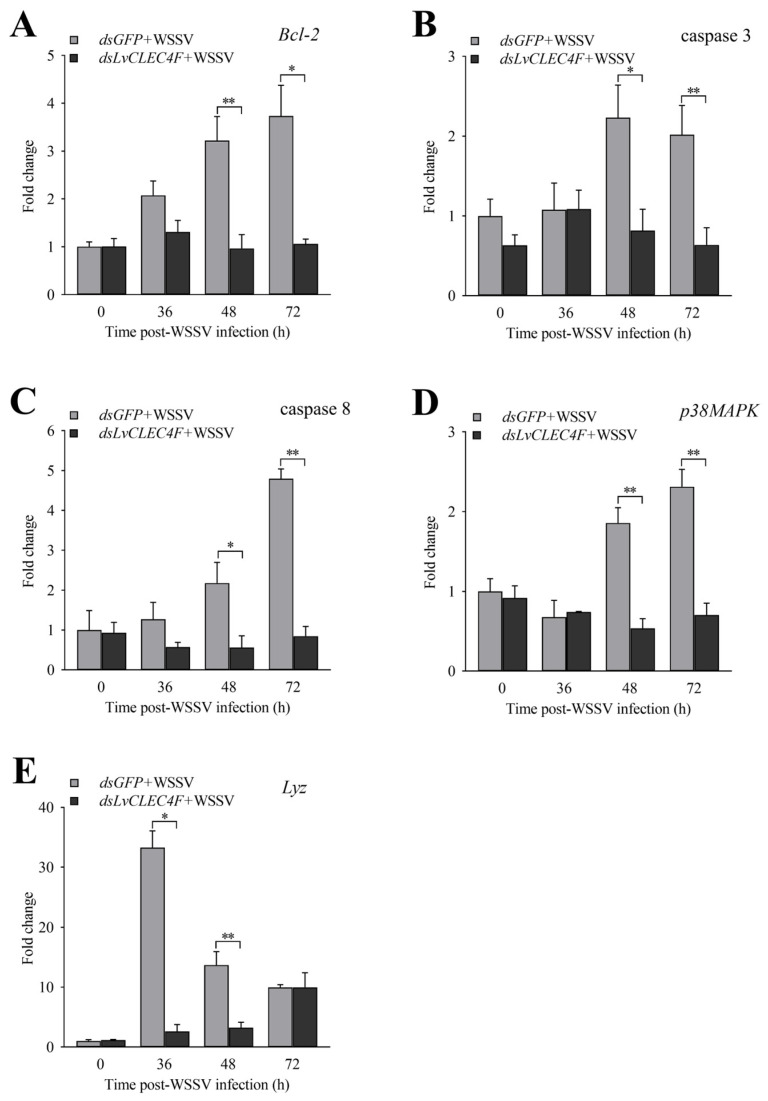
Expression profiles of immune-related genes after knocking down *LvCLEC4F*. (**A**) The expression level of *Bcl-2* at different time points post-WSSV infection after knocking down *LvCLEC4F*. (**B**) The expression level of caspase 3 at different time points post-WSSV infection after knocking down *LvCLEC4F*. (**C**) The expression level of caspase 8 at different time points post-WSSV infection after knocking down *LvCLEC4F*. (**D**) The expression level of *p38MAPK* at different time points post-WSSV infection after knocking down *LvCLEC4F*. (**E**) The expression level of *Lyz* at different time points post-WSSV infection after knocking down *LvCLEC4F*. *: *p* < 0.05, **: *p* < 0.01.

RT-PCR was used to detect the expression levels of genes related to innate immunity in *L*. *vannamei* after knocking down *LvCLEC4F*. The expression levels of *Bcl*-*2* in the *dsLvCLEC4F*+WSSV group at 36, 48, and 72 h were approximately 0.63-fold, 0.30-fold (*p* < 0.01), and 0.28-fold (*p* < 0.05), respectively, compared to those in the *dsGFP*+WSSV group (Figure 8A). The expression levels of caspase 3 in the *dsLvCLEC4F*+WSSV group at 36, 48, and 72 h were approximately 1.01-fold, 0.37-fold (*p* < 0.05), and 0.32-fold (*p* < 0.01), respectively, compared to those in the *dsGFP*+WSSV group (Figure 8B). The expression levels of caspase 8 in the *dsLvCLEC4F*+WSSV group at 36, 48, and 72 h were approximately 0.46-fold, 0.26-fold (*p* < 0.05), and 0.18-fold (*p* < 0.01), respectively, compared to those in the *dsGFP*+WSSV group (Figure 8C). The expression levels of *p38MAPK* in the *dsLvCLEC4F*+WSSV group at 36, 48, and 72 h were approximately 1.09-fold, 0.28-fold (*p* < 0.01), and 0.30-fold (*p* < 0.01), respectively, compared to those in the *dsGFP*+WSSV group (Figure 8D). The expression levels of *Lyz* in the *dsLvCLEC4F*+WSSV group at 36, 48, and 72 h were approximately 0.08-fold (*p* < 0.05), 0.23-fold (*p* < 0.01), and 1.00-fold, respectively, compared to those in the *dsGFP*+WSSV group (Figure 8E).

## 4. Discussion

CTLs serve as crucial PRRs and immune modulators and play a significant role in innate immune responses [22]. Studies have confirmed that CTLs exhibit pathogen-binding and agglutinating activities. Three CTLs (*Mj*LecA, *Mj*LecB, and *Mj*LecC) of *M*. *japonicus* bind to viral envelope proteins, preventing WSSV infection of hemocytes [23]. The CTLs (*Pc*Lec-1 and *Pc*Lec-2) of crayfish participate in immune responses against bacterial and viral infections [24,25]. A shrimp CTL displays a bacterial agglutination ability [26]. The present study cloned *LvCLEC4F* from *L*. *vannamei*. *LvCLEC4F* contains a 492 bp ORF encoding 163 amino acids, comprising a signal peptide structure and CRD (Figure 1). The CRD of CTLs can bind to carbohydrate molecules on the pathogen surface, triggering immune responses [27]. LvCTL 4.2 in *L*. *vannamei* was a C-type mannose-binding lectin with a CRD containing a mutated mannose-binding motif that could facilitate WSSV pathogenesis [20]. A crayfish CTL contains CRD that can bind to *Vibrio* and WSSV [22].

The present study found that *LvCLEC4F* is expressed in the hepatopancreas, gill, muscle, and eyestalk of healthy *L*. *vannamei*. The expression level of *LvCLEC4F* was the highest in the hepatopancreas compared to all other organs and tissues examined (Figure 4). A previous report stated that *LvCTL7* expression levels in *L*. *vannamei* were higher in the hepatopancreas, muscle, gill, and eyestalk, but lower in the brain, epidermis, thoracic ganglion, intestine, heart, and hemocytes [6]. In the current study, the expression level of *LvCLEC4F* in the hepatopancreas was greatly influenced by WSSV infection, showing significant upregulation (Figure 5A). This was consistent with the upregulation of hepatopancreas CTL of a related shrimp following bacterial and WSSV challenges [28].

Knocking down the expression of *LvCLEC4F* in *L*. *vannamei* via RNAi resulted in a significant decrease in the WSSV viral load (Figure 6B) and a significant increase in the survival rate of *L*. *vannamei* (Figure 6C), indicating that *LvCLEC4F* might promote WSSV infection. Studies have reported that soluble CTLs capture and present virions to cell surface receptors to facilitate viral infection. The replication of WSSV was inhibited after the knockdown of a CTL expression of a related shrimp [16]. Moreover, the WSSV viral load in *L*. *vannamei* significantly decreased after knocking down *Pv*DnaJC16 [29]. Like the lectin family, the toll receptor family is a group of conserved PRRs that primarily control the initiation of innate immune responses. Silencing Toll2 significantly increases the survival rate of shrimp post-WSSV infection and reduces the viral load, suggesting that Toll2 can promote WSSV infection in shrimp [30]. Based on the above results, it was speculated that *LvCLEC4F* could promote the replication of WSSV and affect the survival rate of *L*. *vannamei*.

To investigate the reason for the improved immunity of *L*. *vannamei* after knocking down *LvCLEC4F*, the hemolymph agglutination in *L*. *vannamei* following WSSV infection after knocking down *LvCLEC4F* was observed. The expression levels of genes related to the regulation of apoptosis, signaling and cellular stress response, and immune defense were analyzed. The results indicated an apparent trend of weakened hemolymph aggregation compared to that in the *dsGFP*+WSSV group after knocking down *LvCLEC4F* (Figure 7). LvLdlrCTL exhibits agglutination activity against bacteria and fungi and potentiates the phagocytosis of hemocytes [31]. Relevant signaling pathways, including the apoptotic pathway, and other immune pathways, can exert immune functions post-WSSV infection [32,33,34]. Experimental studies have shown that apoptosis induced by WSSV infection constitutes a pivotal host defense response against viral infections [35]. In the present study, the expression of genes in the apoptotic pathway, primarily *Bcl-2* (Figure 8A), caspase 3 (Figure 8B), and caspase 8 (Figure 8C), showed a significant downward trend after knocking down *LvCLEC4F*. Additionally, a significant decrease in WSSV viral load was detected after knocking down *LvCLEC4F*. The findings were similar to a previous study, which demonstrated that intramuscular injection of r*Lv*HSP70 in *L*. *vannamei* effectively extended the survival rate of WSSV-infected shrimp and led to a decreased WSSV viral load. Furthermore, a significant reduction in the expression of apoptosis-related genes was observed [36].

The expression of gene *p38MAPK* (Figure 8D) showed a significant downward trend after knocking down *LvCLEC4F*. *p38MAPK* was a vital gene induced in response to WSSV infection in a shrimp [34]. In this study, the expression of *Lyz* showed a significant downward trend following the knockdown of *LvCLEC4F* (Figure 8E). It was consistent with a previous report that after knocking down *MrLec*, the expression of lysozyme 2 was downregulated in a freshwater shrimp post-*Vibrio* challenge [37]. AMPs play crucial roles in innate immune defense responses [38]. Previous studies have also shown that knocking down *LvLdlrCTL* leads to the upregulation or downregulation of many immune effector genes in shrimp post-WSSV infection [31].

## 5. Conclusions

In conclusion, a *LvCLEC4F* has been cloned from *L*. *vannamei*. The expression profiles of *LvCLEC4F* in the hepatopancreas, gill, muscle, and eyestalk of *L*. *vannamei* were significantly altered post-WSSV infection. After knocking down *LvCLEC4F*, the survival rate of *L*. *vannamei* significantly increased, and the WSSV viral load significantly decreased. In addition, the hemolymph agglutination phenomenon was weakened. Finally, the expression of genes related to the regulation of apoptosis, signaling and cellular stress response, and immune defense (*Bcl-2*, caspase 3, caspase 8, *p38MAPK*, and *Lyz*) were significantly downregulated. Overall, *LvCLEC4F* was an important receptor gene that could promote WSSV replication and affect the survival rate of *L*. *vannamei*.

## Figures and Tables

**Table 1:** The sequences of primers and probes used in this study.

Primer or Probe Name	Sequences (5′–3′)
LvCLEC4F-F	CTTGAAGGCAACACAAACGC
LvCLEC4F-R	TTTGCATTCCTCAACTAAAACTACA
qLvCLEC4F-F	TTCGTGCTCCTGCTGTCCTT
qLvCLEC4F-R	CAAACTCTCTGGGCGTTGGGT
18S-F	TATACGCTAGTGGAGCTGGAA
18S-R	GGGGAGGTAGTGACGAAAAAT
WSSV-F	TGGTCCCGTCCTCATCTCAG
WSSV-R	GCTGCCTTGCCGGAAATTA
WSSV-probe	AGCCATGAAGAATGCCGTCTATCACACA
Bcl-2-F	GCTATGTGTCCTTTGTGGCT
Bcl-2-R	TGAACTTGGCAATGGTAACTG
caspase3-F	AGTTAGTACAAACAGATTGGAGCG
caspase3-R	CGGTCCTTGTGGACAGACAG
caspase 8-F	GGCGACAAGATGAGGCAA
caspase 8-R	CAGGGTGAGGGAGAGAAAACT
p38MAPK-F	GTCGGCTCGCAACTACATAC
p38MAPK-R	CCGTTACACGCCTTTCACT
Lyz-F	ACTGGTGCGGAAGCGACTA
Lyz-R	GGCGGATAGTCTCGGCG
dsGFP-Fi	GCGTAATACGACTCACTATAGGCATCTTCTTCAAGGACGACGG
dsGFP-Ri	GCGTAATACGACTCACTATAGGAGTTCACCTTGATGCCGTTCT
LvCLEC4F-Fi	GCGTAATACGACTCACTATAGGCGCGCAAAATGATGTTCTTCGT
LvCLEC4F-Ri	GCGTAATACGACTCACTATAGGTGGAGCGCTACAATCATAATCAAA

## Data Availability

The original contributions presented in the study are included in the article; further inquiries can be directed to the corresponding author/s.

## References

[1] Hou C., Song W., Yuan H., Hu N., Tan B., Zhang S. (2022). Comparative transcriptome analysis revealed that dietary zymosan-A improved the immunity of *Penaeus vannamei* by regulating the TLR signaling pathway. Aquaculture.

[2] Lightner D.V. (2012). Global transboundary disease politics: The OIE perspective. J. Invertebr. Pathol..

[3] Lightner D.V. (2011). Virus diseases of farmed shrimp in the Western Hemisphere (the Americas): A review. J. Invertebr. Pathol..

[4] Chen J., Li Z., Hew C. (2007). Characterization of a novel envelope protein WSV010 of shrimp white spot syndrome virus and its interaction with a major viral structural protein VP24. Virology.

[5] Verbruggen B., Bickley L., van Aerle R., Bateman K.S., Stentiford G.D., Santos E.M., Tyler C.R. (2016). Molecular Mechanisms of White Spot Syndrome Virus Infection and Perspectives on Treatments. Viruses.

[6] Luo J., Chen Y., Huang Y., Feng J., Yuan Y., Jian J., Cai S., Yang S. (2023). A novel C-type lectin for *Litopenaeus vannamei* involved in the innate immune response against *Vibrio* infection. Fish Shellfish Immunol..

[7] Hoffmann J.A., Kafatos F.C., Janeway C.A., Ezekowitz R.A.B. (1999). Phylogenetic Perspectives in Innate Immunity. Science.

[8] De Gregorio E., Spellman P.T., Rubin G.M., Lemaitr B. (2001). Genome-wide analysis of the *Drosophila* immune response by using oligonucleotide microarrays. Proc. Natl. Acad. Sci. USA.

[9] Wang X., Wang J. (2013). Pattern recognition receptors acting in innate immune system of shrimp against pathogen infections. Fish Shellfish Immunol..

[10] Sancho D., Reis e Sousa C. (2012). Signaling by Myeloid C-type Lectin Receptors in Immunity and Homeostasis. Annu. Rev. Immunol..

[11] Drickamer K. (1993). Evolution of Ca^2+^-dependent Animal Lectins. Prog. Nucl. Res. Molec. Biol..

[12] Drickamer K. (1988). Two Distinct Classes of Carbohydrate-recognition Domains in Animal Lectins. J. Biol. Chem..

[13] Rogers N.C., Slack E.C., Edwards A.D., Nolte M.A., Schulz O., Schweighoffer E., Williams D.L., Gordon S., Tybulewicz V.L., Brown G.D. (2005). Syk-Dependent Cytokine Induction by Dectin-1 Reveals a Novel Pattern Recognition Pathway for C Type Lectins. Immunity.

[14] Smith L.C., Azumi K., Nonaka M. (1999). Complement systems in invertebrates. The ancient alternative and lectin pathways. Immunopharmacology.

[15] Van Vliet S.J., García-Vallejo J.J., van Kooy Y. (2008). Dendritic cells and C-type lectin receptors: Coupling innate to adaptive immune responses. Immunol. Cell Biol..

[16] Wang X., Xu Y., Xu J., Zhao X., Wang J. (2014). Collaboration between a soluble C-type lectin and calreticulin facilitates white spot syndrome virus infection in shrimp. J. Immunol..

[17] Li Y., Pan L., Yu J. (2022). The injection of one recombinant C-type lectin (LvLec) induced the immune response of hemocytes in *Litopenaeus vannamei*. Fish Shellfish Immunol..

[18] Luo M., Yang L., Wang Z., Zuo H., Weng S., He J., Xu X. (2019). A novel C-type lectin with microbiostatic and immune regulatory functions from *Litopenaeus vannamei*. Fish Shellfish Immunol..

[19] Sun J., Lan J., Zhao X., Vasta G.R., Wang J. (2017). Binding of a C-type lectin’s coiled-coil domain to the Domeless receptor directly activates the JAK/STAT pathway in the shrimp immune response to bacterial infection. PLoS Pathog..

[20] Huang Y.H., Kumar R., Liu C.H., Lin S.S., Wang H.C. (2022). A novel C-type lectin LvCTL 4.2 has antibacterial activity but facilitates WSSV infection in shrimp (*L*. *vannamei*). Dev. Comp. Immunol..

[21] Durand S.V., Lightner D.V. (2002). Quantitative real time PCR for the measurement of white spot syndrome virus in shrimp. J. Fish Dis..

[22] Chen D., Meng X., Xu J., Yu J., Meng M., Wang J. (2013). PcLT, a novel C-type lectin from *Procambarus clarkii*, is involved in the innate defense against *Vibrio alginolyticus* and WSSV. Dev. Comp. Immunol..

[23] Song K., Li D., Zhang M., Yang H., Ruan L., Xu X. (2010). Cloning and characterization of three novel WSSV recognizing lectins from shrimp *Marsupenaeus japonicus*. Fish Shellfish Immunol..

[24] Wang X., Zhang H., Li X., Zhao X., Wang J. (2011). Characterization of a C-type lectin (PcLec2) as an upstream detector in the prophenoloxidase activating system of red swamp crayfish. Fish Shellfish Immunol..

[25] Zhang X., Wang X., Sun C., Zhao X., Wang J. (2011). C-type lectin from red swamp crayfish *Procambarus clarkii* participates in cellular immune response. Arch. Insect Biochem. Physiol..

[26] Alenton R.R.R., Koiwai K., Nakamura R., Thawonsuwan J., Kondo H., Hirono I. (2019). A Hint of Primitive Mucosal Immunity in Shrimp through *Marsupenaeus japonicus* Gill C-Type Lectin. J. Immunol..

[27] Mahla R.S., Reddy M.C., Prasad D.V., Kumar H. (2013). Sweeten PAMPs: Role of Sugar Complexed PAMPs in Innate Immunity and Vaccine Biology. Front. Immunol..

[28] Zhang X., Xu W., Wang X., Mu Y., Zhao X., Yu X., Wang J. (2009). A novel C-type lectin with two CRD domains from Chinese shrimp *Fenneropenaeus chinensis* functions as a pattern recognition protein. Mol. Immunol..

[29] Jaree P., Somboonwiwat K. (2023). DnaJC16, the molecular chaperone, is implicated in hemocyte apoptosis and facilitates of WSSV infection in shrimp. Fish Shellfish Immunol..

[30] Wang C., Zhang H., Zhu J., Liu H., Yang Y., Sun B., Wu T., Zhang Y., Yao D. (2023). The transcription factor CEBP homolog of *Penaeus vannamei* contributes to WSSV replication. Fish Shellfish Immunol..

[31] Liang Z., Yang L., Zheng J., Zuo H., Weng S., He J., Xu X. (2019). A low-density lipoprotein receptor (LDLR) class A domain-containing C-type lectin from *Litopenaeus vannamei* plays opposite roles in antibacterial and antiviral responses. Dev. Comp. Immunol..

[32] Dostert C., Jouanguy E., Irving P., Troxler L., Galiana-Arnoux D., Hetru C., Hoffmann J.A., Imler J.L. (2005). The Jak-STAT signaling pathway is required but not sufficient for the antiviral response of drosophila. Nat. Immunol..

[33] Cai J., Huang Y., Wei S., Huang X., Ye F., Fu J., Qin Q. (2011). Characterization of p38 MAPKs from orange-spotted grouper, Epinephelus coioides involved in SGIV infection. Fish Shellfish Immunol..

[34] He Y., Yao W., Liu P., Li J., Wang Q. (2018). Expression profiles of the p38 MAPK signaling pathway from Chinese shrimp *Fenneropenaeus chinensis* in response to viral and bacterial infections. Gene.

[35] Leu J.H., Lin S.J., Huang J.Y., Chen T.C., Lo C.F. (2013). A model for apoptotic interaction between white spot syndrome virus and shrimp. Fish Shellfish Immunol..

[36] Janewanthanakul S., Supungul P., Tang S., Tassanakajon A. (2020). Heat shock protein 70 from *Litopenaeus vannamei* (*Lv*HSP70) is involved in the innate immune response against white spot syndrome virus (WSSV) infection. Dev. Comp. Immunol..

[37] Huang X., Li W., Jin M., Ma F.T., Huang Y., Shi Y.R., Zhao L.L., Feng J.L., Ren Q., Wang W. (2016). Single CRD containing lectin from *Macrobrachium rosenbergii* (*MrLec*) participates in innate immunity against pathogen infections. Fish Shellfish Immunol..

[38] Chen S.L., Li W., Meng L., Sha Z.X., Wang Z.J., Ren G.C. (2007). Molecular cloning and expression analysis of a hepcidin antimicrobial peptide gene from turbot (*Scophthalmus maximus*). Fish Shellfish Immunol..

